# Exploiting activation radiation from neutron tomography reveals the hidden elemental composition of 3D art objects for free

**DOI:** 10.1038/s41598-024-80047-4

**Published:** 2024-11-22

**Authors:** Yueer Li, Sara Creange, Zhou Zhou, William Southworth, Arie Pappot, Lambert van Eijck

**Affiliations:** 1 Delft University of Technology, Faculty of Applied Sciences, Delft, 2629JB-15 The Netherlands; 2 Rijksmuseum, Amsterdam, 1071 XX The Netherlands; 3 Center for Neutron Science and Technology, School of Physics, Sun Yat-Sen University, Guangzhou, 510275 China; 4 Rijksmuseum, Amsterdam, 1070 DN The Netherlands

**Keywords:** Imaging, Neutron activation, Gamma spectroscopy, Non-destructive, Quantification, Cultural heritage, Bronze, Applied physics, Nuclear physics, Characterization and analytical techniques, Imaging techniques

## Abstract

Neutron tomography is gaining popularity particularly in cultural heritage research, for non-destructively analysing the inner structure of bulk metal artefacts, such as bronzes, but the induced temporary decay radiation is often considered as a drawback. However, this delayed gamma-emission can be put to good use: by performing gamma spectroscopy after neutron tomography, the interior elemental composition of artefacts can be obtained “for free”. Inspired by this, we propose a ray-tracing approach to non-invasively quantify both interior geometry and elemental composition using only a single neutron tomography experiment. This strategy aligns well with both the aim for efficient use of neutron beam time and the expectation from curators and conservators for minimal neutron irradiation. Here, we outline the core principle of this method, demonstrate the extent of its quantification capability on bulk objects of known composition by fusing neutron tomography and delayed-gamma spectroscopy data sets. We also showcase its practical application on an ancient solid-cast Indonesian bronze statuette, by which we gain insights into how the pristine inner bronze segregated into a different composition than the surrounding shell. Similarly, the method allows us to quantify the composition of a hidden offering in the statuette that consecrates the bronze for worship purposes.

## Introduction

**Fig. 1:**
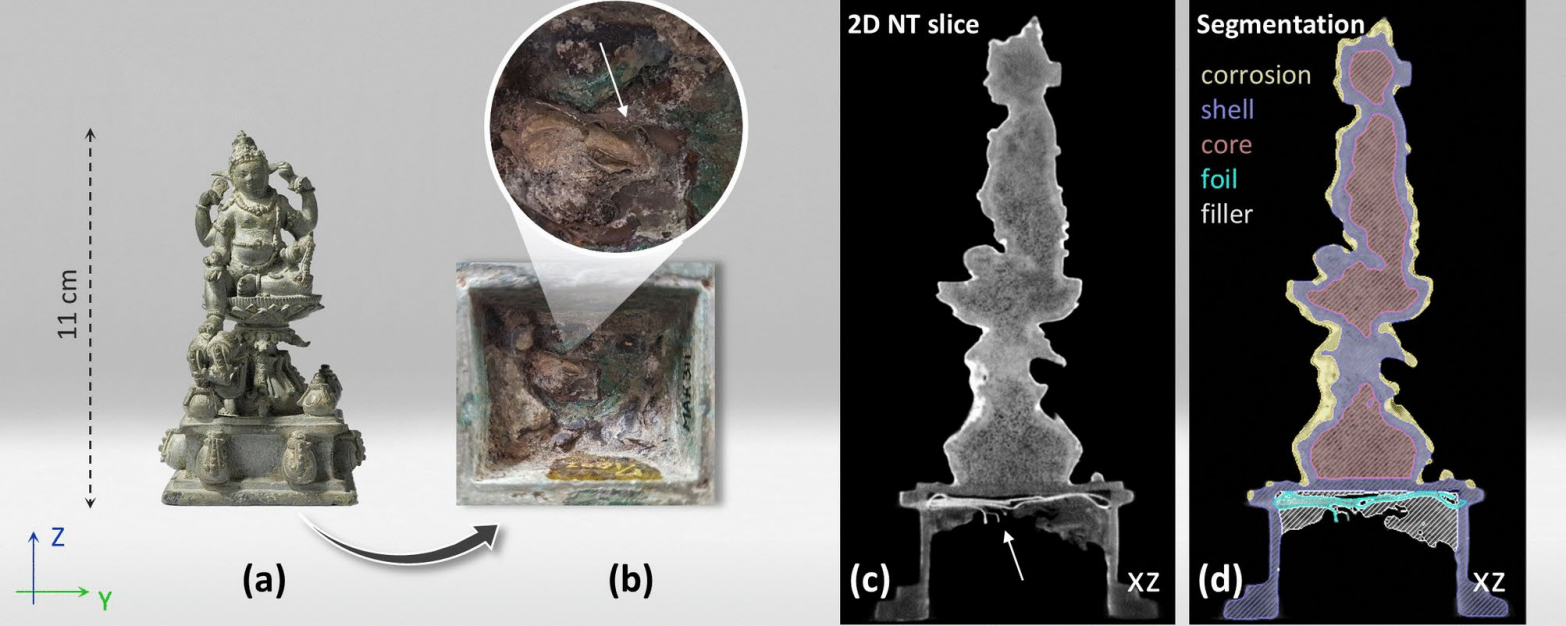
Neutron tomography of the Kuvera statuette. (**a**) Bronze representation of Kuvera or Jambhala, the god of prosperity in Hinduism and Buddhism. This statuette is in the Asian collection of the Rijksmuseum Amsterdam (object number: AK-MAK-311, c. 850–930)^11^. (**b**) A bottom view of its pedestal where a small part of the foil sticks out (white arrow) of the filler material meant to seal this consecration deposit. (**c**) A vertical slice of the reconstructed NT model: the bulk bronze appears rather porous. Underneath the baseplate of the pedestal, a folded foil is visible with high brightness. Underneath the foil, an irregular filling material is visible that mostly covers the foil. (**d**) Segmentation of different regions according to the grey tones. The regions can be tentatively assigned to different material compositions.

The addition of an appropriate amount of tin to molten copper can make copper stronger, harder, and easier to cast; this discovery, made thousands of years ago, led to what we now call the Bronze Age. Since then, copper alloyed with tin (and other trace elements) has been widely used for casting objects of various sizes for various purposes^1–5^. Thanks to the chemical stability of bronze, many such objects have been well preserved and have become cultural legacies of ancient civilizations, with significance beyond their original functions. An example of such artefacts is a solid bronze statuette from Indonesia (Fig. 1a), crafted to honour the god of prosperity, known as Kuvera in Hinduism or Jambhala in Buddhism. This statuette, only 11cm in height, impresses with its intricate details, such as jewellery and a mongoose in its left-hand spewing pearls or coins, vividly depicting an image of wealth and demonstrating the casting craftsmanship of the artist(s) of its time. Notably, the statuette’s hollow pedestal, cast as one with the solid figure, is filled with greenish and brownish materials, with a yellowish metal foil sticking out, indicating a consecration deposit sealed by the filler (Fig. 1b). Consecrating statues with the addition of such small objects is common in several regions, including the Himalayas and Indonesia^6,7^. To gain deeper insights into the craftsmanship and cultural customs of the ancient society, as well as to assess the current state of corrosion in the artefact without causing damage, neutron tomography (NT)^8^ was performed to visualize the internal structure, followed by gamma spectroscopy (GS) to obtain elemental information on the entire object. The main aim of the iterative method that we present in this paper is to obtain quantitative information on the bulk of (heritage) materials, through data fusion of the NT and GS experimental data.

Compared to X-rays, neutrons offer visibility of both light and heavy elements, even when the bronze is obscured by thick corrosion layers. Fig. 1 shows the Kuvera statuette, a NT slice, and a segmentation highlighting different regions based on their grey tones. Despite a thin layer of superficial bronze corrosion (yellow segment), the statuette appears to be in good condition overall. The outer bronze “shell” (lavender purple) appears more solid than the inner bronze “core” (rose pink), which displays higher porosity, probably due to the casting process where the shell solidified first, forcing gases to move to the core (and outside). As anticipated, a folded foil (cyan) showing a high grey tone was discovered within the pedestal, mostly covered by filler material (white). Since the foil shows a notably higher grey tone compared to the main bronze, considering the associated ritual customs^9^, it is reasonable to hypothesize that the foil is made of gold or silver (based on the neutron attenuation coefficients (cm^−1^) of Au (6.23) > Ag (4.04) > Cu (1.07) > Sn (0.21)^10^). Even though it is possible to distinguish distinct bronze compositions (corrosion, shell, core) and the hidden foil, it is not possible to directly draw conclusions solely from NT to answer questions, such as which bronze composition was used for this statue, which corrosion products are associated with it, as well as which material was used for the foil offering in the pedestal. In heritage science, such information is usually obtained from XRF analysis, which has limited capability to quantify the elemental composition and probes only the outer micrometres of the object that may have undergone changes due to segregation during casting, degradation, alterations, or conservations during its lifetime. In this context, neutron-based elemental composition characterization methods are ideally used to obtain the missing elemental information of the (pristine) inner materials.

Neutron-based techniques such as prompt-gamma activation analysis (PGAA)^12,13^, instrumental neutron activation analysis (INAA)^14,15^, and neutron resonance capture analysis (NRCA)^16,17^ can non-invasively determine the interior elemental composition of bulk objects, such as bronze statuettes. These methods exploit the penetration power of neutrons and their induced gamma-rays, providing a quantitative way to analyse element masses in the entire (bulk) metal artefacts. PGAA is considered a routine analytical method at neutron research facilities and is being actively developed further, such as PGAI-NT^18^, ibNAA^19^, and T-PGAA^20^. Nevertheless, PGAA facility is not as common as INAA, as it has higher requirements for detector efficiency and shielding for both neutrons and gamma rays. In this manuscript, we demonstrate that even without the infrastructure of PGAA on-site, one can use delayed-emitted gamma rays from the induced radioactivity to obtain quantitative information on the bulk composition in a non-invasive manner. We argue that the temporary radioactivity of the object after NT can be exploited for this purpose, through ray-tracing simulations.

Our method, similar to INAA, measures delayed radioactive decay through gamma spectroscopy, but the neutron activation is performed in an NT experiment. The only “infrastructure” requirement for measuring such gamma radiation is basically a HPGe detector in a low-gamma-background environment (typically, away from the beam line). INAA is well-known for its highly accurate and precise elemental analysis of homogeneous powders in milligram quantities^21^. However, for large objects like bronze statuettes (of the order of centimetres), attenuation effects of penetrating neutrons and gamma rays become significant^22^. Therefore, corrections for self-attenuation effects of neutrons and gamma photons are essential for accurate quantification^15^. The mainstream approach of applying INAA to bulk objects involves determining factors for correcting these effects, either by theoretical modelling (based on the Beer–Lambert law), by Monte Carlo modelling assuming objects with simple geometry or homogeneity^23,24^, or by using a priori available composition information of a test portion sampled from the object^25,26^. In the case of ancient cast bronzes, these calculations become tedious due to irregular shapes as well as internal inhomogeneities. Our method allows the incorporation of such complexities into the analysis through ray-tracing simulations. Although the temporary radioactivity induced by NT is often considered a disadvantage in cultural heritage research, it can be put to good use: by performing GS after NT, the elemental composition of the interior of the object can be extracted to some extent. A method, which quantitatively and non-invasively determines both structure and composition of the interior of an object, is of great value in heritage science.

**Fig. 2:**
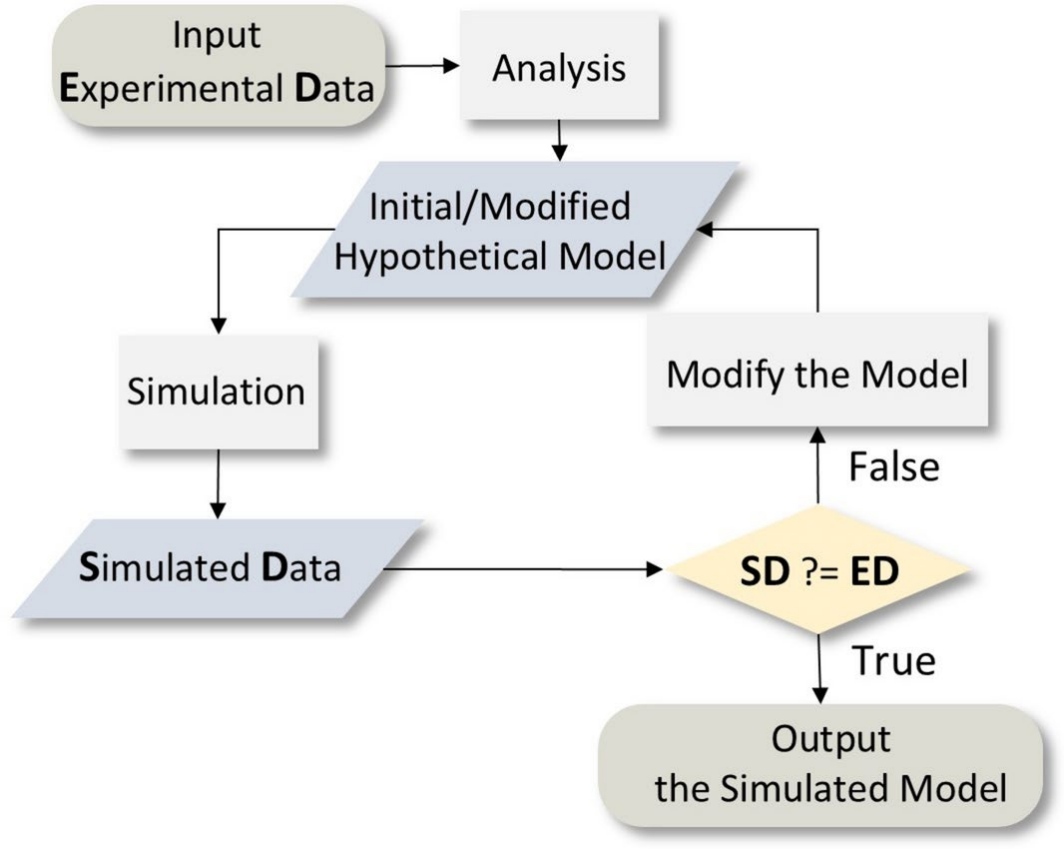
The iterative method. The process is divided into four steps: (1) analysing the experimental NT and GS data; (2) making a hypothetical model of material structure and composition for a simulation; (3) reproducing NT and GS data using Monte Carlo simulations; (4) comparing the simulated data with the experimental data. If the simulations match the experimental data, end the process; otherwise repeat steps (2)–(4).

While NT and GS solely provide spatial and elemental resolution, respectively, their data sets are quantitative, which in turn presents a unique opportunity to fuse structure and elemental information. For instance, if the foil (Fig. 1b) is made of pure gold, its mass can be determined from the volumetric data of NT. Similarly, the mass can be obtained from the intensity of radioactive decay from gold through GS (if the attenuation effects are considered properly). If these two calculations yield the same mass of gold, it is a likely justification for the hypothesis; if not, the hypothesis can be revised until the two results align. Inspired by this concept, we designed an iterative approach (Fig. 2) based on ray-tracing simulations to fuse NT and GS data sets, thereby localizing the interior materials and quantifying their elemental compositions.

Our aim is to reproduce both NT and GS data sets using Monte Carlo ray-tracing simulations, where the attenuation correction effects of neutrons and gamma rays can be properly accounted for, regardless of the shape complexity. The core principle involves: (1) running multiple simulations with varied elemental composition assumptions, and (2) comparing the simulated data with experimental data to determine the composition that best matches the experimental data. Moreover, once the elemental composition is determined, our method can further predict how long an object remains radioactive, which is a common concern in cultural heritage research involving NT. In this paper, we outline the method and demonstrate its precision by benchmarking the simulations using two objects with known compositions. Additionally, we showcase the potential of the method by applying it to the precious Kuvera statuette (Fig. 1a).

## Methods

To implement the data fusion algorithm described above, we first perform NT and (subsequent) GS on the object. These experimental data sets are then simulated through ray-tracing calculations of both experiments, using 3D models extracted from the NT reconstruction. The initial elemental composition assigned to the 3D model is based on the GS data. Subsequently, the composition is adjusted in an iterative process to reproduce both the gamma spectra as well as the neutron tomography projections. All experiments and simulations described in this paper were conducted at the TU Delft Reactor Institute in the Netherlands at the FISH beam line^27^. The maximum capture flux for this thermal NT beam line is 7e^6^ n/cm^2^/s.

### Object geometry from neutron tomography

The three-dimensional model (Fig. 1c), reconstructed using Octopus software^28^, represents both the atomic density as well as the neutron absorption and scattering cross-sections of local compositions^29^. Utilizing this data, materials with different grey tones can be segmented using Avizo software, as shown in Fig. 1d. These segmented volumes are subsequently converted into surface models that define the geometries in the simulations.

### The first composition from gamma spectroscopy

The radioactive decays induced by NT are measured as a series of gamma spectra, where gamma intensities exponentially decrease over time according to the decay constants of activated isotopes (see the gamma spectra collected from the GS experiment of the Kuvera statuette in Supplementary Fig. S1 online). The mass of element $j$, determined from a spectrum peak centred at energy $E_{k}$, can be calculated from an experiment with irradiation time $t_{i}$, delayed time $t_{d}$, and measurement time $t_{m}$ using the absolute method^30^:

1 $$m_{{j,E_{k} }} = \frac{A}{{\frac{{\theta \cdot N_{av} }}{{M_{a} }} \cdot \Phi_{0} \cdot N^{*} \cdot \sigma \cdot \frac{1}{\lambda } \cdot \left( {1 - e^{{ - \lambda \cdot t_{i} }} } \right) \cdot e^{{ - \lambda \cdot t_{i} }} \cdot \left( {1 - e^{{ - \lambda \cdot t_{i} }} } \right) \cdot \Gamma \cdot \varepsilon \cdot G^{*} }}$$

where $A$ is net peak area in the gamma spectrum collected during the measurement. $\theta$, $\lambda$, and $M_{a}$ are the natural abundance, decay constant, and molar mass of the target isotope, respectively, while $N_{av}$ is the Avogadro constant. $\Phi_{0}$ is the flux of incident neutrons, and $\sigma$ is the isotopic cross-section of thermal neutrons. $\Gamma$ is the gamma-ray abundance, and $\varepsilon$ is the full energy photopeak efficiency of the detector, calibrated by using a point source. $N^{*}$ and $G^{*}$, the correction factors for neutrons and gamma rays, are used for compensating the losses from self-attenuation effects. These factors can be approximately determined via the Beer–Lambert law, by assuming the object is a homogeneous cuboid with a volume equivalent to the tomography model. The elemental mass composition throughout the object can be determined by analysing each photon peak. It is important to note that not all elements can be analysed by GS. For instance, lead (Pb) is invisible in GS.

### Simulation of experiments using Monte Carlo method

After assigning each segment of the object an initial elemental composition in the ray-tracing models, the particle beam as well as the interactions between particles and matters are simulated using Monte Carlo stochastics^31^. In practice, we utilized TOPAS MC^32^ to perform Geant4-based Monte Carlo simulations^33^. The default “Shielding” physical model of particle-matter interactions was applied in the simulations. Due to the incomplete atomic de-excitation database within Geant4, a modification of the Geant4 databases was needed to produce gamma rays from metastable isotopes. For instance, gamma rays from Sn-125m were introduced by replacing the half-life and decay scheme of Sn-125 in the database (G4ENSDFSTATE and RadioactiveDecay, respectively) by those of Sn-125m. In fact, because the corresponding cross section affects both the gamma yield as well as the neutron attenuation in the simulation, this value was not modified, and we implemented the correct gamma yield in the simulations output, only.

In the simulations, the neutron beam was modelled as a parallel beam with Gaussian position distributions in both horizontal and vertical directions as illustrated in Supplementary Fig. S2 online. To simulate the accumulation effects of nuclei activation, the intensity of the neutron beam was modelled as multiple pulses, spread over the irradiation time. The neutron camera and gamma detector were modelled as surface scorers with dimensions matching those of the experimental neutron scintillator and the germanium crystal inside the gamma detector. These surface scorers record information about incident particles, including type, energy, arrival time, and arrival position, which could be converted into neutron projections or gamma spectra. Typically, one simulation of both data sets requires several hours on an 8-CPU workstation, depending strongly on the model complexity. By simulating approximately 100 million neutron trajectories through the object, the resulting neutron projections and gamma spectra can be compared with experimental data. Based on the differences between the simulated and experimental data, the composition of the segments in the model is adjusted to improve the match.

### Comparing simulations with experiments

#### Comparison of neutron projections

Firstly, flat-field correction^34^ needs to be applied to both the experimental and simulated projection pictures $P$, and then their residual $\Delta P$ is calculated pixel by pixel through:

2 $$\Delta P = {\text{ABS}}\left( {\frac{{P_{simu} - P_{expt} }}{{P_{expt} }}} \right)$$

To individually assess the residual of each material segment $l$, the differences are further analysed by using its binary mask image $M$, the median of its residual is determined via:

3 $$\Delta P_{l} = {\text{MED}}\left( {\Delta P \cdot M_{l} } \right)$$

#### Comparison of radioactive decays

The simulated gamma photon intensity $I_{simu}$ of energy $E_{k}$ needs to be corrected by considering the differences between the experiment and the simulation in terms of the number of impinging neutrons as well as the detector efficiency:

4 $$I _{{simu,E_{k} }}^{\prime} = I_{{simu,E_{k} }} \cdot \frac{{\Phi_{expt} }}{{\Phi_{simu} }} \cdot \frac{{\varepsilon_{{expt,E_{k} }} }}{{\varepsilon_{simu} }}$$

where $\Phi_{expt}$ and $\Phi_{simu}$ are the neutron fluxes in the experiment and simulations, respectively. $\varepsilon_{simu}$ is the geometric efficiency of the simulated detector without gamma-energy resolution, and $\varepsilon_{{expt,E_{k} }}$ represents the full energy photopeak efficiency of experimental detector for gamma energy $E_{k}$.

Then, the simulated gamma intensity can be compared to the experimental gamma intensity, without additional scaling factors, and their residual is defined as:

5 $$\Delta I_{{E_{k} }} = {\text{ABS}}\left( {\frac{{I _{{simu,E_{k} }}^{\prime} }}{{I_{{expt,E_{k} }} }} - 1} \right)$$

where the quotient $\frac{{I _{{simu,E_{k} }}^{\prime} }}{{I_{{expt,E_{k} }} }}$ is determined using Orthogonal Distance Regression (ODR) calculations implemented in the SciPy python package^35^.

#### Residuals based iterative procedure

The total residual ${\Delta }_{total}$, combining residuals from neutron projections of $L$ segmented materials as well as residuals from $K$ gamma spectrum energy peaks, is determined by:

6 $$\Delta_{{{\text{total}}}} = \mathop \sum \limits_{l}^{L} \Delta P_{l} + \mathop \sum \limits_{k}^{K} \Delta I_{{E_{k} }}$$

An ideal simulation would yield a residual of 0, implying that the closer the residual is to 0, the more likely the assumed composition in the simulation matches the actual composition. Following this principle, the elemental composition can be updated iteratively according to ${\Delta }_{total}$ calculated in current (and previous) simulations, aiming to reduce ${\Delta }_{total}$ in the next comparison.

## Materials and experiments

**Fig. 3:**
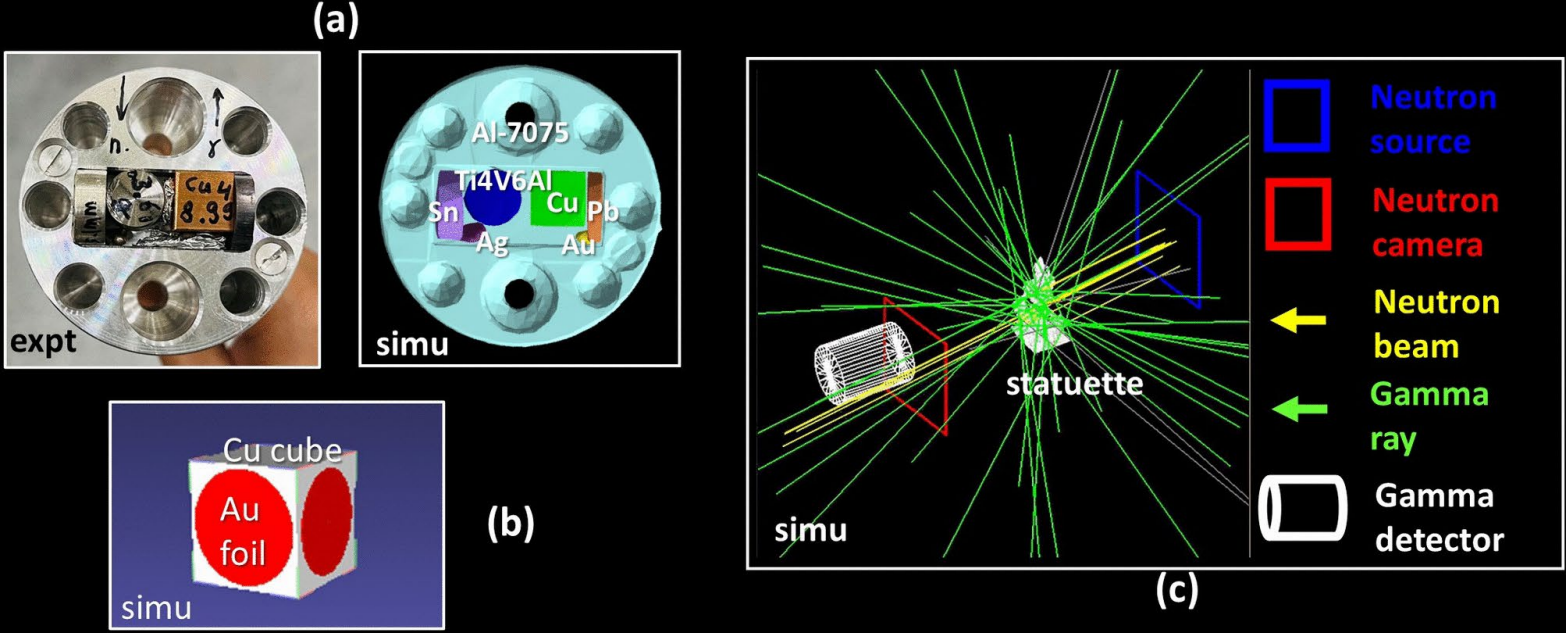
Objects used in the experiments: (**a**) Cylindrical object (CYL): top views of the CYL in the experiment (left) and simulation (right); (**b**) Cubic object (CUBE): CUBE model in the simulation; (**c**) Screenshot of the simulation of the NT and GS experiments on the Kuvera statuette (Fig. 1).

First, the validation of Geant4-based simulations is demonstrated by a set of six known objects contained within an aluminium cylinder (CYL) (Fig. 3a), for which both the NT and GS experimental data are reproduced. Then, our method is demonstrated using a simple composition of a copper cube with three thin gold foils (CUBE) (Fig. 3b). The iteration routine can determine the mass of the CUBE, solely from the gamma spectrum. Lastly, the method is applied to “data-fuse” the NT and GS experiments on the Kuvera bronze statuette, as shown in Fig. 3c, where both the NT and GS components are shown simultaneously.

### CYL

The CYL is comprised of a large aluminium (Al-7075) cylinder, along with six small objects: a copper cube, a tin cylinder, a lead cylinder, a titanium alloy (Ti6Al4V) cylinder, a gold bead, and a silver nugget. To measure short half-life isotopes the object was first irradiated for 20 min in the neutron beam, thereby enabling a better signal-to-noise ratio in the GS detection. Then, NT on the CYL was done for 2.5 h, during which 501 projections from different orientations were collected. Subsequently, GS was performed to detect the long half-life isotopes. The model (the 3D geometries from the NT reconstruction, and their known composition) was then used in the simulations to reproduce NT and GS data. These simulated data were then compared with the experimental data to assess the accuracy and precision of the simulations in reproducing the experimental results.

### CUBE

The demonstration of the iterative approach is done with a CUBE consists of a 1 cm^3^ copper cube and three gold foils, each with a thickness of 0.05 mm. The foils were positioned individually against the side of the copper cube and wrapped together using an aluminium foil. The CUBE was irradiated by neutrons for 2.5 h without rotation. Then, the radioactive decay was measured by GS, from which the masses of gold and copper were approximately determined using Eq. (1). These initial mass estimates served as the starting point for subsequent iterative simulations aimed at optimizing the masses to better match the actual masses. This optimization process was implemented through TopasOpt^36^.

### Kuvera statuette

The experiment procedure of the Kuvera, similar to the CYL experiment, involved an irradiation of 30 min followed GS measurements for short half-life isotopes. Then, NT was performed using 1500 orientations of the Kuvera in the beam, resulting in an irradiation of 23.5 h. Following this, GS was performed starting approximately 30 min after the NT and lasting for 3 days.

The NT model was segmented into the bronze (consisting of corrosion, shell, and core), the foil, and the filler (Fig. 1d) based on their grey tones. To assign an initial composition to each of these 5 segments, the masses per element were estimated from the GS data, based on Eq. (1), using (initial, coarse) shielding corrections that approximate the geometry as a 2 × 3 × 11 cm^3^ cuboid. These masses were then assigned to the segments (core and shell: {Cu, Sn, As, Sb, Co, Mn}, corrosion: {H, Na, Cl, Cu, Sn, As, Sb, Co, Mn}, foil: {Au, Ag}, filler: {Sn, Cu}).

The first approximation is that elements As, Sb, Co and Mn (with concentrations less than 1 wt%) hardly contribute to the neutron attenuation, while H (present only in corrosion layer) has a very strong contribution. Moreover, H, Na, and Cl are assumed to be associated with the corrosion layer only, while the other trace elements are assumed to be evenly distributed over the bronze. According to the ancient Indonesian tradition^7^ and our NT analysis (as illustrated in Supplementary Fig. S3 online), tin and bronze were used in the filler segment, which we confirmed with XRF measurements (Supplementary Table S1 online). XRF also provides an approximate Sn/Cu ratio of the corrosion layer. These segments, with their corresponding composition, were used as an initial hypothetical model for use in an iterative optimization procedure of composition determination. For practical reasons, we demonstrate this procedure for a range of compositions that were ‘manually’ set, rather than determined by an iteration routine.

The segments “wrap” each other: the core is enveloped by the shell, which is covered by the corrosion layer. Matching the simulation projections to the experimental data will imply that any composition changes in one segment will affect the analysis of the other two. To circumvent this effect, the optimization workflow was designed in a specific analysing order from outer segment to inner segment, progressing from the corrosion layer to the shell and finally to the core. Once the composition of the outer segment is determined, it is fixed to determine the composition of neighbouring inner segment(s).

Since the grey value of the corrosion layer is dominated by H, firstly, the ratio of H to (Sn/Cu) of the simulation is matched using the experimental projections. With this corrosion composition fixed, the Sn/Cu ratio of the shell is matched likewise. Lastly, this procedure is repeated for the core, filler and the foil. When comparing the simulated GS using these compositions to the experimental spectra, it turns out that the Sn and Cu GS signals are slightly less than the experimental data (with a mismatch of 31% and 10%, respectively). The most obvious modification to then make is the Sn/Cu ratio of the corrosion layer (which was inferred from XRF). This order, comparing NT projections from outside to inside and then comparing GS, allows to determine a self-consistent set of compositions after a few iterations. With the bronze compositions fixed, finally, the Sn/Cu composition of filler and the Au/Ag composition of the foil are determined.

## Results and discussion

### Method validation

**Fig. 4:**
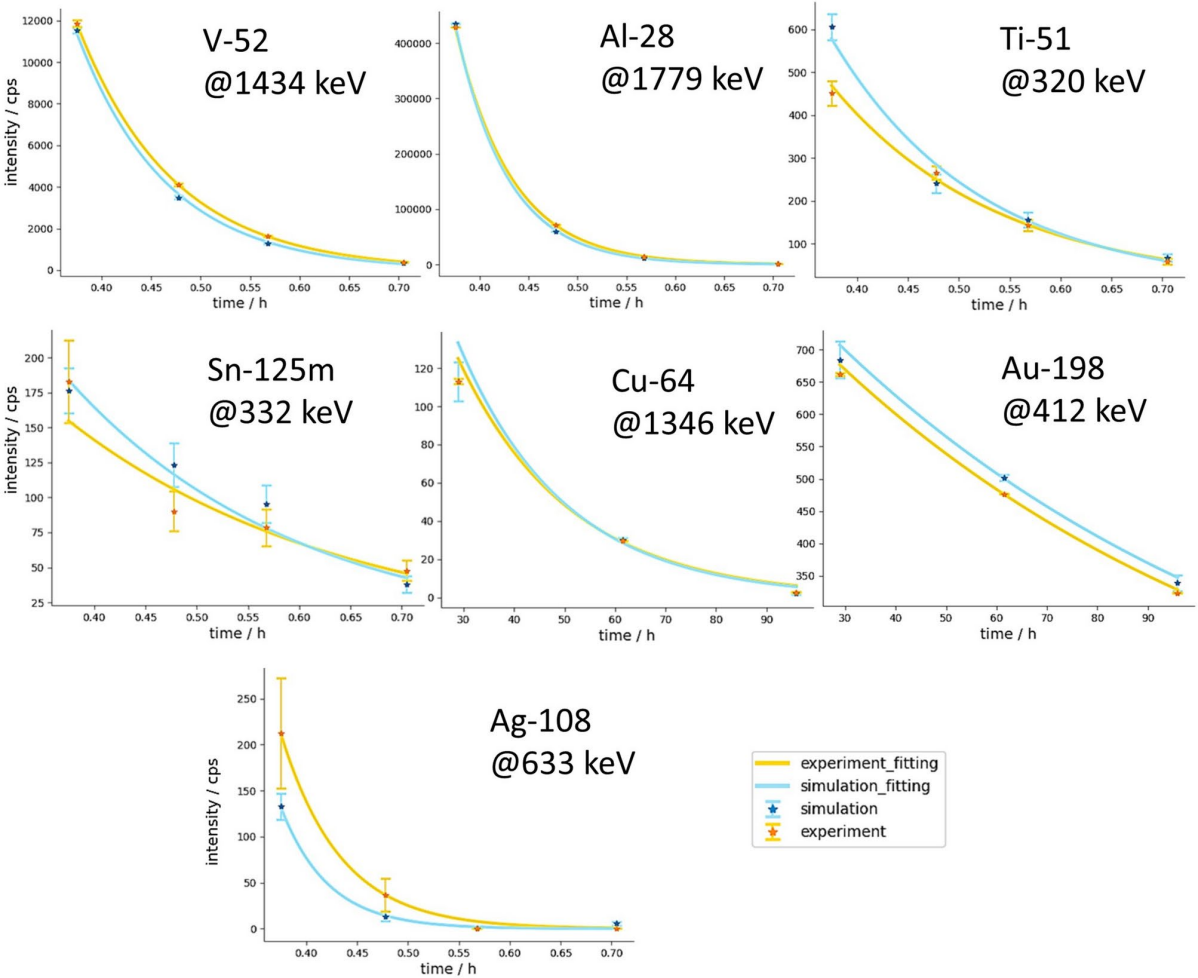
Correspondence of the time-dependent simulated and experimental gamma intensity for the different isotopes of the CYL. Notably, all these simulated gamma intensities contain no scaling factor.

Figure 4a shows the agreement in the gamma spectroscopy between the simulation and the experiment for the known objects (CYL), achieved without the need for scaling or calibrations. For all the studied isotopes, the gamma intensity was simulated to be of the same order of magnitude as that observed in the experiment, and it decayed with a similar rate as that in the experiment. The most noticeable deviations are that of the Ti-51 and Ag-108, two short half-life components. Their intensities were affected by the much stronger Al-28 decay and the spectral background generated by this (~ 230 g) cylinder. In the Supplementary Fig. S4 online, we demonstrated the improved correspondence when the aluminium cylinder is absent during the irradiation. Although the agreement is not perfect, the comparison indicates that the gamma spectroscopy data can be quantitative predicted by simulation of the experiments (NT irradiation and GS). Additionally, the simulations based on the Geant4 databases are capable to predict the gamma intensities effectively.

**Fig. 5:**
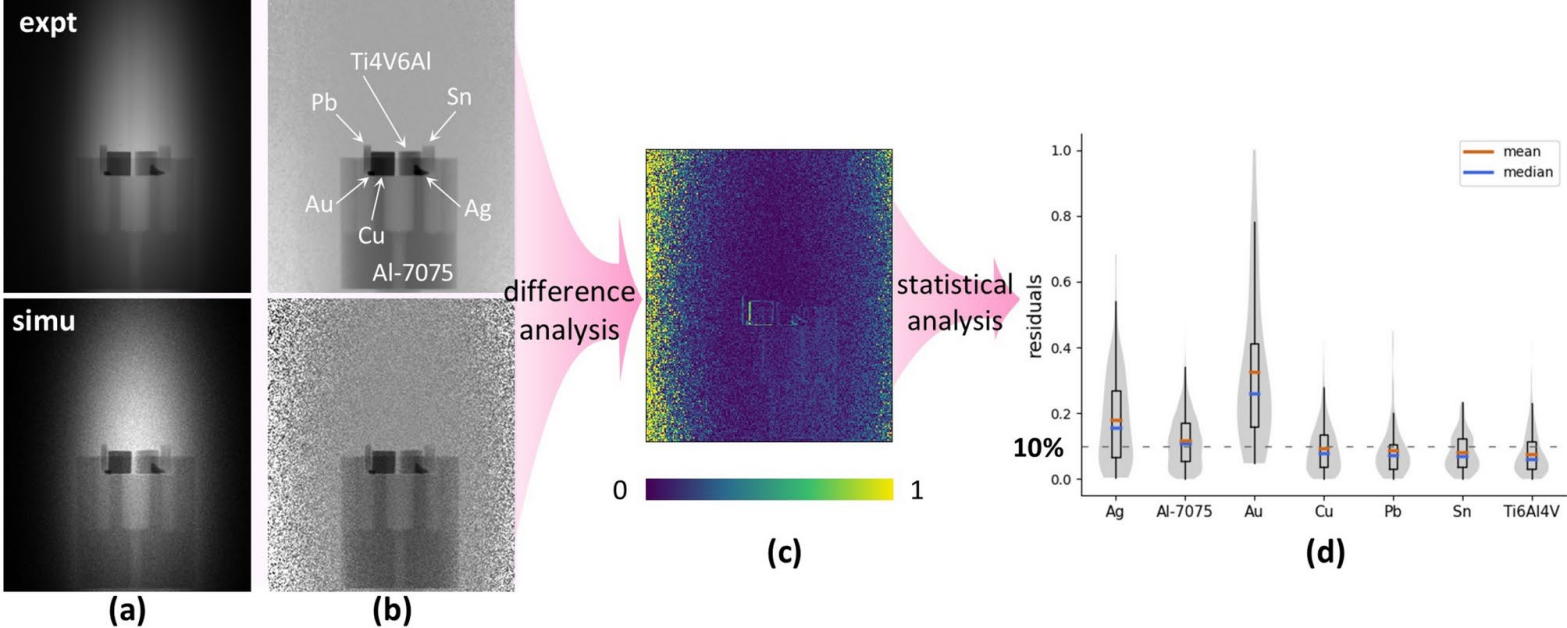
Comparison of simulated and experimental NT data for the CYL experiment. (**a**) Experimental and simulated NT projections at the same orientation of the CYL in the beam. (**b**) Normalized experimental and simulated projections for comparison. (**c**) Residual of the comparison, determined through Eq. (2). A perfect agreement would result in all pixels being dark blue. (**d**) Statistics results of NT pixel residuals for each material segment. The distribution of data points is shown in grey.

Since the GS method lacks spatial resolution, the GS result reports the per-isotope intensity integrated over the entire cylinder. As a results, the measured copper signal includes contributions from both the Cu cube and the Al-7075 cylinder, making the Cu mass quantification as a whole, rather than independently. However, by applying material masks in the analysis of the NT projections, the neutron attenuation effect of each material can be observed and quantified independently (Fig. 5). We exploit this for Pb, which is not detected by GS but exhibits a clear signature in the NT projections, as shown in Fig. 5a,b. The residuals of NT projections were quantified per pixel, as shown in Fig. 5c, with most disagreements observed at the rim of the figure and the rim of objects. Further statistical analysis of the NT residuals per material segment is presented in Fig. 5d. Notable data outliers, mainly caused by simulated particles with 30 times lower intensity than those in the experiment, increased the residual uncertainty. Nonetheless, the median values remained representative of the pixel residuals across the entire material area. Especially small or transparent objects can pose challenges in obtaining accurate segmentation, thereby limiting the accuracy of our method. For instance, the segmented dimensions of the tiny Au bead, Ag nugget, and the (transparent) Al cylinder were respectively +26%, +15%, and −10% off from their physical dimensions, resulting in their relatively larger residuals of 33%, 18%, and 12%, respectively. Despite these discrepancies, good correspondence between the simulated model and the actual object was achieved, with the median of the residuals for other elements is below 10%.

Combining the results from GS and NT provides a more comprehensive understanding of the discrepancies arising from each element within each material of the CYL (see details in Supplementary Table S2 online). Ag and Au, with the smallest weight and volume, exhibited the largest total residual of 60% and 38%, respectively. This indicates relatively high simulation uncertainty associated with the analysis of elements present in smaller quantities. For the remaining elements, the simulated data closely matched the experimental data, suggesting that the complexities of the interactions between objects and particles were effectively accounted for in the simulations. While certain factors, such as neutron beam divergence and detector efficiency, were not fully incorporated into the simulations, leading to slightly less accurate simulated results, a good overall agreement with the experimental data (overall difference ≤ 20%, except Ti, which is 23%) was achieved. Future work will involve modelling more realistic neutron beam and detector systems to enhance the accuracy of the simulations.

The experiment with the cube (CUBE) aimed to validate the iterative approach. Table 1 demonstrates a significant improvement in mass calculation accuracy after performing 50 iterations, compared to the mass initially calculated by GS (Supplementary Video S1 online). This outcome confirms the feasibility of achieving accurate mass composition quantification using iterations with Monte Carlo simulations. Although this experiment is based only on GS data, coupled with the findings from the CYL experiment, we believe that the addition of the comparison “simu-expt” of the NT projections will further improve the composition quantification through imposing an extra constraint. For heritage science, where the composition is not known, this methods allows to determine the composition, based on minimization of the differences between simulation and experiment.

**Table 1:** Elemental composition of the CUBE from iterations.

Material	Weighted mass (g)	Initial calculated mass before iterations (g)	Relative error of calculated mass compared to weighted mass (%)	Final calculated mass after 50 iterations (g)	Relative error of calculated mass compared to weighted mass (%)
Cu cube	9.06	10.59	17	8.33	8
Au foils	0.19	0.24	27	0.19	0

### Analysis of the Kuvera statuette

**Fig. 6:**
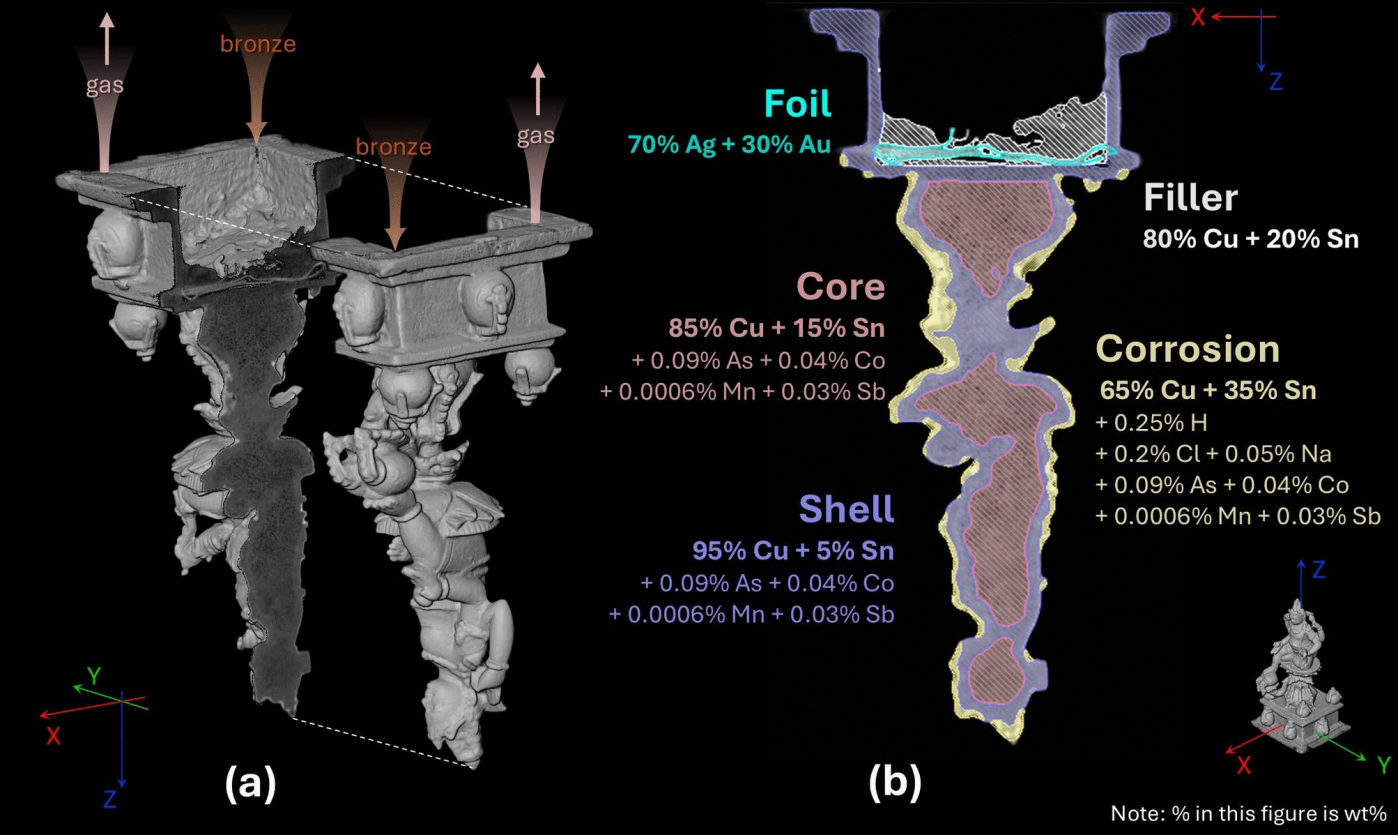
(**a**) Possible casting process of the Kuvera statuette. (**b**) The quantified elemental composition of each material segment of this statuette. See Supplementary Table S3 for the summary of the analytical elemental masses.

As shown in Fig. 1, the solid bronze consists of three regions, of which the corrosion layer was absent when the statue was cast. The analysis of the 3D NT model suggests that this solid bronze statuette was made via the lost-wax casting technique, as shown in Fig. 6a. During casting, the clay mould was positioned upside down, and the molten bronze was poured into the mould through a system of pouring channels and vents, of which we can see remnants at the bottom of the pedestal walls. This process, combined with the slow cooling of the liquid bronze, led to the segregation of the bronze into the distinct (solid) shell and (porous) core region. It is apparent that the core is particularly porous, and our new data fusion algorithm allows to quantify that the composition of this region is different from that of the shell region, as shown in Fig. 6b (see detailed simulation results in Supplementary Fig. S5 online). The shell bronze (5 wt% Sn) solidified first, expelling some of the tin towards the core (15 wt% Sn) and possibly the outer surface. Based on the assumption of homogeneity of molten bronze, we infer that the original bronze material prepared by the artist contained 85–95 wt% Cu and 5–15 wt% Sn, characteristic of low-tin bronze statuettes.

The corrosion analysis revealed a significant association with hydrogen, chlorine, and sodium. Despite hydrogen being undetected by GS, its notable neutron attenuating effects were measured by neutron projection, suggesting a certain amount of hydrogen presence within the corrosion layer (see the quantification results in Supplementary Fig. S5). Additionally, chlorine and sodium were identified in the corrosion layer, indicating the presence of potential corrosion products such as copper chlorides and chalconatronite^37^. These three corrosion-associated elements were present in moderate amounts, implying that only a small amount of material was corroded. This observation aligns well with the analytical results from NT slices and visual inspections. By comparing the composition of the corrosion bronze (65 wt% Cu) with that of the pristine bronze in the core and the shell (approximately 90 wt% Cu), we tentatively conclude that the corrosion layer has undergone decuprification, which may have occurred during burial.

Contrary to our initial hypothesis that the foil was pure gold, based on its yellowish appearance, our results surprisingly indicate that the foil comprises primarily silver (70 wt%) with a small amount of gold (30 wt%). This finding could reflect an intentional choice of alloy, perhaps to economize on gold for making a silver-gilt foil, or it might have been an unintended result of recycling precious metals. For the filler material, although its composition was initially identified as an alloy of Cu and Sn, further structural analysis (Supplementary Fig. S3 online) suggests that it is more likely a mixture of low-tin bronze foil(s) and pure tin. The bronze foil(s) could have been used as a support to fix the deposit during tin casting and/or as a spacer to prevent the deposit from getting contamination of the solder material (Sn), or they might have served other unknown ritual purposes.

## Conclusion

We established a non-destructive method to quantify both the internal elemental composition and structure of bulk objects using only a single neutron tomography experiment. Specifically, this method involves performing delayed-gamma spectroscopy after neutron tomography to gain composition information “for free” and utilizing ray-tracing simulations to quantitatively fuse these NT and GS data sets in an iterative procedure. Even if the object consists of distinct materials rather than one homogeneous alloy, our method allows to assign a plausible composition of each part.

This method has been validated in two ways, utilizing objects of known composition. The simulation procedure can reproduce both the neutron projections as well as the gamma intensity with a total residual of less than 20%. Since it is based on segmentation of the NT-reconstructed 3D model, the method is limited by the spatial resolution of the NT experiment. Likewise, the analysis of the GS data is limited by the beam flux and the signal-to-noise ratio can be diminished by the Compton background of the different elements in the object, as we demonstrate in determining silver from the Ag-108 signal, hindered by the Al-28 background. By assigning specific elements to sub-volumes, an iterative process can determine the masses of these sub-volumes with an uncertainty of less than 10%. This method was then successfully applied to a bronze Kuvera statuette—a complex analytical case with corrosion, phase segregation, and a consecration deposit. Our method allowed to quantify the interior elemental composition of this statuette from a single NT experiment, providing potential insights into the (technical) skills, technological and material choices of local craftsmen, as well as the ritual customs of ancient societies.

Despite not achieving perfect correspondence to the experimental data sets and being constrained by limited computational power, this work represents a pioneering step forward in multimodal neutron techniques, expanding the applications of both neutron tomography and gamma spectroscopy, through data fusion of these two modalities. In addition, our approach only requires a single HPGe detector and an 8-CPU desktop computer to add the extra “element-dimension” to the 3D model of the neutron tomography and is therefore widely applicable. Materials science of heritage objects is notoriously challenging because of the heterogeneity of the pristine object as well as its centuries-long (un-) intentional modifications. The ability to non-destructively obtain quantitative structural and compositional information on the hidden (pristine) bulk material of these objects is a great asset for heritage science. We believe that our method offers such value and can be further applied in other cultural heritage research involving objects such as Chinese bi-metallic bronze swords^38^, Van Leeuwenhoek’s microscope^39^, and glasses with multiple colours^40^. We demonstrate that this data fusion method provides new capabilities for beam lines with a neutron flux of ~7e^6^ n/cm^2^/s, with sufficient sensitivity. Furthermore, we expect it can be combined with other characterization techniques, such as X-ray CT^41^, epithermal neutron radiography^42^, and cold neutron radiography/tomography^43^, to broaden its application and to gain more comprehensive insights into the studied object.

## Supplementary Information

Supplementary Video 1.

Supplementary Information.

## Data Availability

The data sets used and/or analysed during the current study are available from the corresponding author on reasonable request.
