# Supplementary material for: Exploiting activation radiation from neutron tomography reveals the hidden elemental composition of 3D art objects for free
Source: Sci Rep. 2024 Nov 22;14:28982. doi: 10.1038/s41598-024-80047-4 (PMC11584630; doi:10.1038/s41598-024-80047-4)
Supplement: Supplementary file 2 — Supplementary Information. [file 41598_2024_80047_MOESM2_ESM.docx]

Supplementary information for

**Exploiting activation radiation from neutron tomography reveals the hidden elemental composition of 3D art objects for free**

Yueer Li^1, *^, Sara Creange^2^, Zhou Zhou^3^, William Southworth^4^, Arie Pappot^2^ & Lambert van Eijck^1^

^1^Delft University of Technology, faculty of Applied Sciences, Delft, 2629JB-15, Netherlands

^2^Rijksmuseum, Amsterdam, 1071 XX, Netherlands

^3^Center for Neutron Science and Technology, School of Physics, Sun Yat-sen University, Guangzhou, 510275, China

^4^Rijksmuseum, Amsterdam, 1070 DN, Netherlands

^*^y.li-32@tudelft.nl


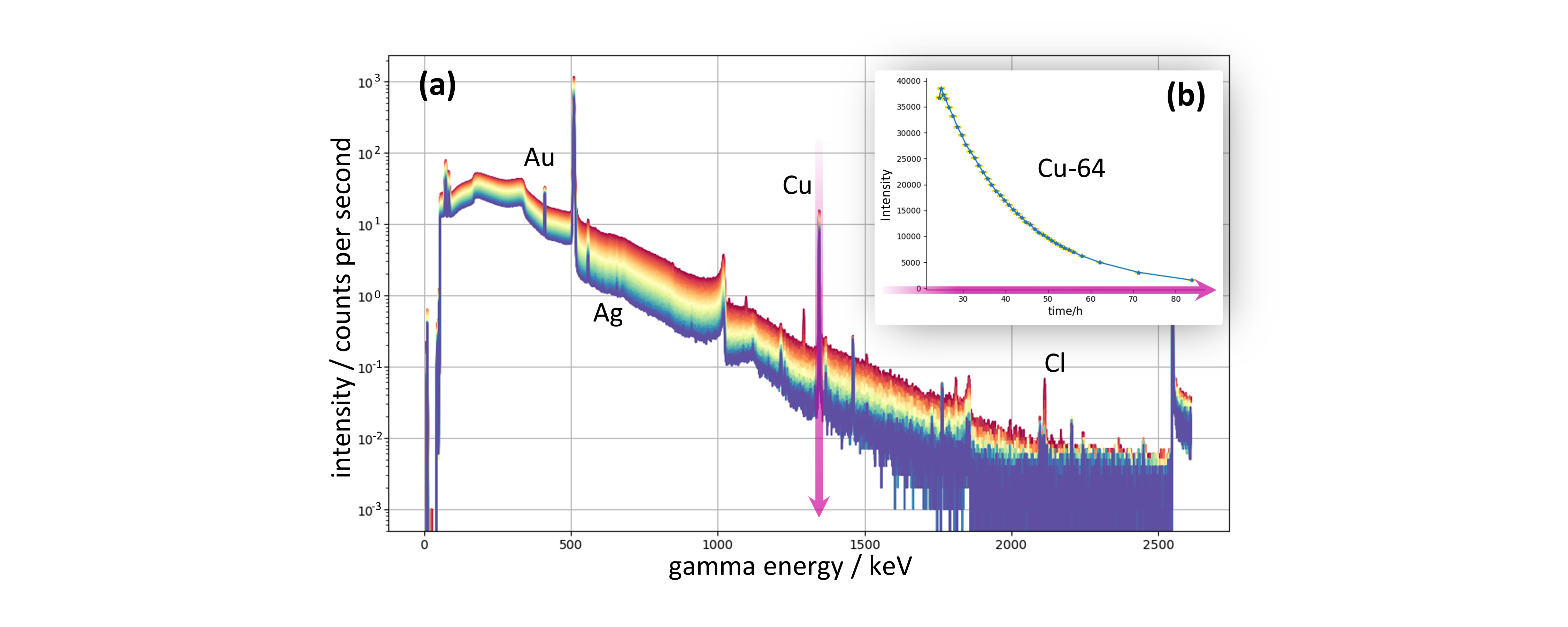


**Supplementary Figure S1.** (**a**) A series of gamma spectra of the Kuvera bronze statuette. (**b**) The radioactive decay at 1345 keV (Cu-64).


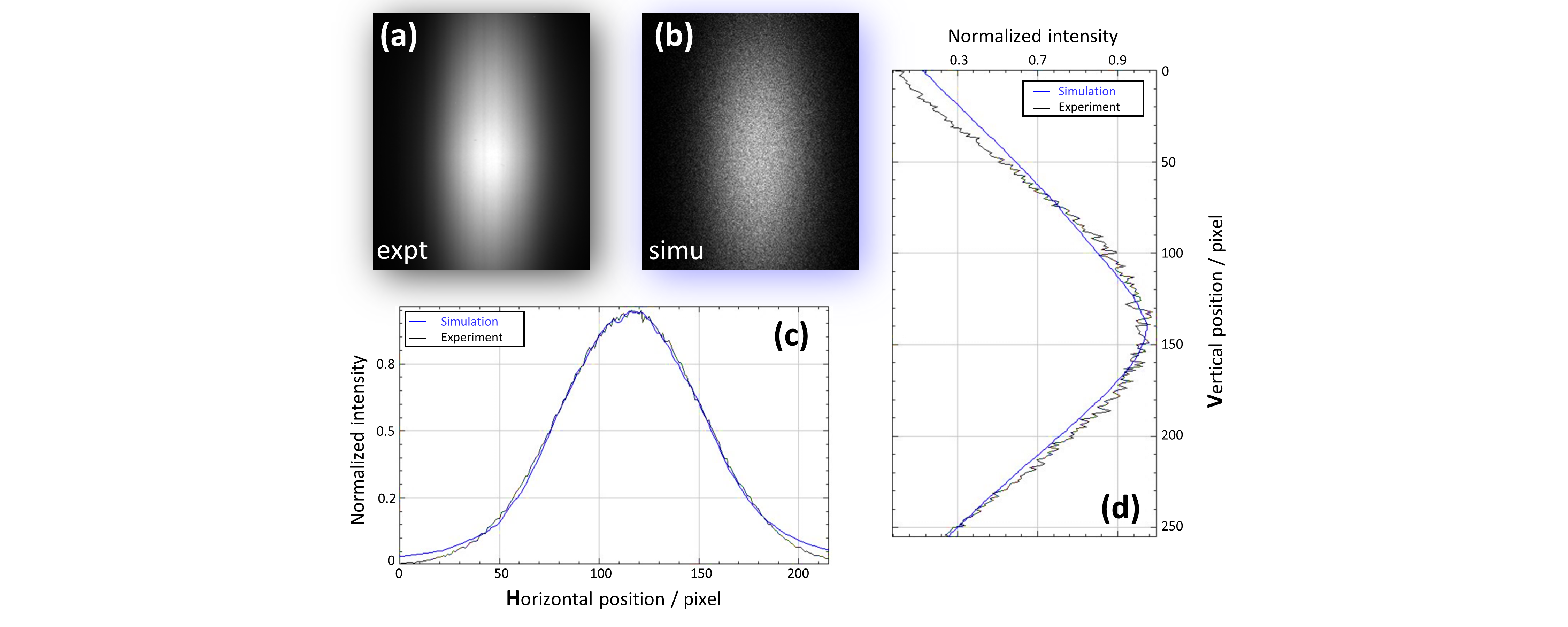


**Supplementary Figure S2.** (**a)** & (**b**) Beam profile in the experiment and simulations, where one binned pixel is 0.5mm. (**c)** & (**d**) By assigning neutron positions with gaussian distributions in both horizontal and vertical directions, the simulated neutron beam well represents the experimental beam.


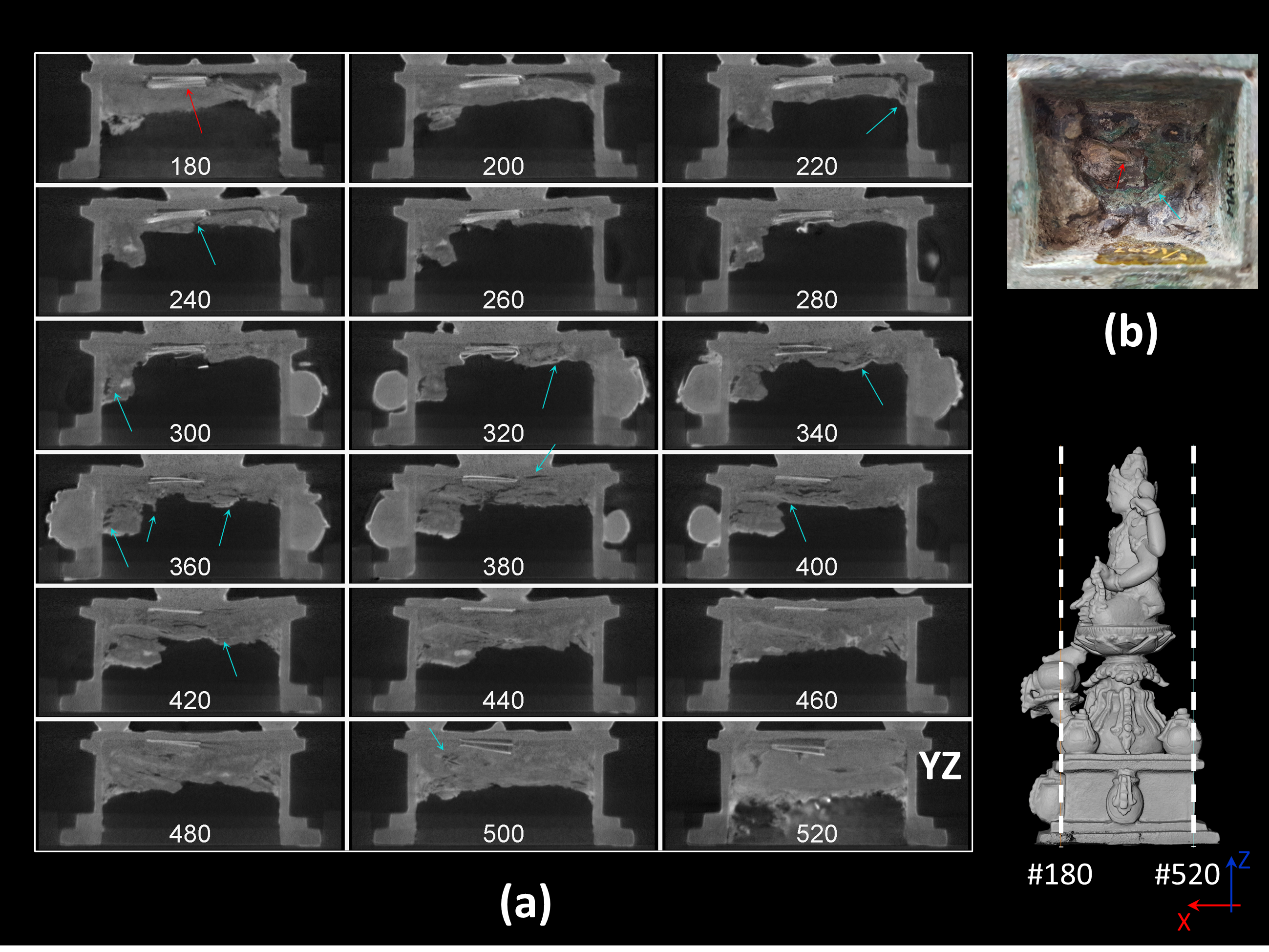


**Supplementary Figure S3.** (**a)** Reconstruction slices of the pedestal of the Kuvera statuette, with a consecration foil (red arrow) hidden inside and part of it sticking out from the filler. The filler is probably a mixture of pure tin (solder material) and bronze foil(s) (cyan arrow). From slice #520, we can see a stacking structure of the tin material, which may caused by multiple tin castings. (**b**) Bottom view of the pedestal, showing the consecration foil (red arrow) and the bronze foil(s) (cyan arrow).


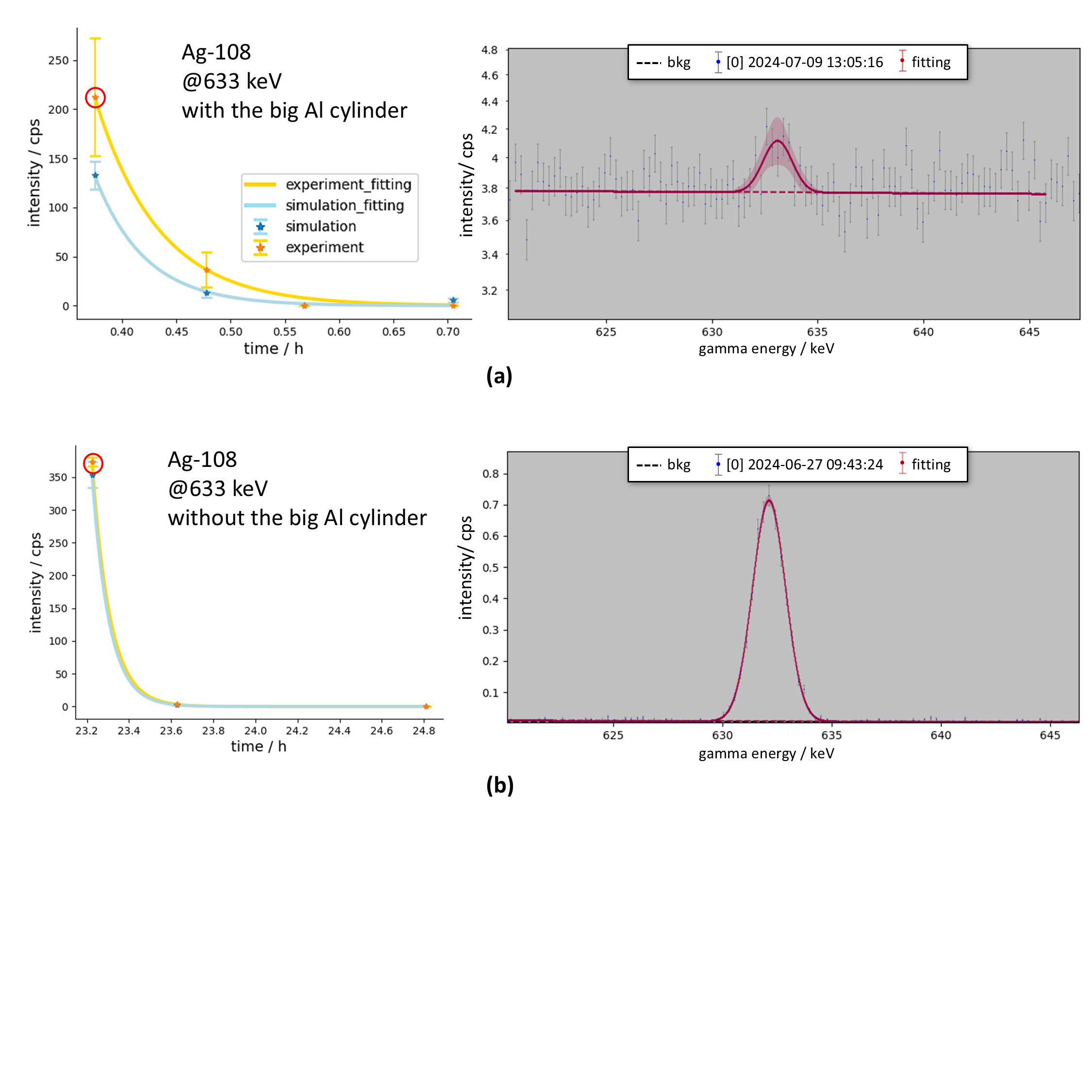


**Supplementary Figure S4.** Comparison of the accuracy of GS simulation for small objects with and without a strong background. Here, we use a silver nugget (from the CYL) as an example to illustrate the effect of a strong spectral background in GS analyses. (**a)** (left) In the CYL experiment, there is a mismatch of 42% between the simulated and experimental gamma intensity from the silver nugget (0.33 g), primarily due to the strong spectral background generated by the (~230 g) aluminium cylinder. (right) The gamma spectrum (blue dots) of the first measurement and the fitted peak (red line) at 633 keV showing a significant uncertainty (shaded area). (**b**) (left) When the silver nugget was irradiated and measured alone in another experiment, the mismatch reduced to 6%, as the fitted peak more accurately represented the gamma data thanks to the improved signal-to-noise ratio. (right) The corresponding gamma spectrum and fitted peak are shown for this scenario.


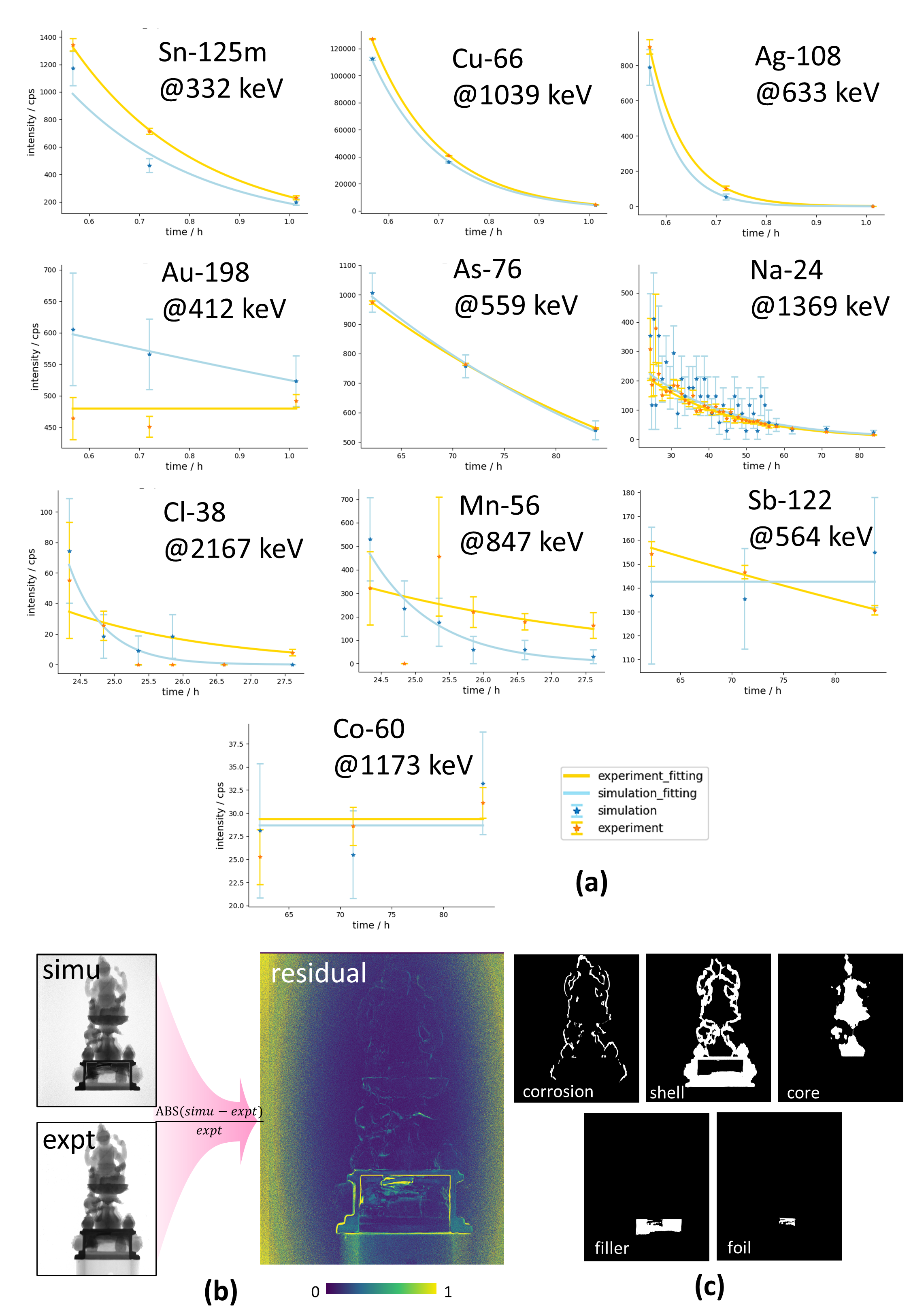


**Supplementary Figure S5.** For the Kuvera statuette, the composition was determined after several iteration of NT and GS simulations. Here we show the results of simulations using this composition. (**a**) Correspondence of the experimental and the simulated gamma intensity for each studied isotope. (**b**) Agreement between the experimental and the simulated NT projection. (**c**) Binary mask of each material segment used in equation (3) to evaluate the NT residual, individually.

**Supplementary Table S1.** XRF results of two different positions of the Kuvera statuette.

| Detection position | Elemental composition [wt%] | | | | | | | | | | | | | |
| --- | --- | --- | --- | --- | --- | --- | --- | --- | --- | --- | --- | --- | --- | --- |
|  | Cu | Sn | Au | Ag | Fe | Pb | Sr | Zn | Bi | Ni | Mo | As | Ti | Co |
| Filler & foil | 14 | 61 | 15 | 7 | 1.23 | 0.66 | 0.36 | 0.09 | 0.03 | 0.03 | 0.01 | \ | \ | \ |
| Bronze corrosion | 77 | 21 | \ | 0.11 | 0.51 | 0.67 | \ | \ | \ | 0.04 | 0.004 | 0.33 | 0.23 | 0.03 |

**Supplementary Table S2.** Summary of the residual between the simulated data and the experimental data for the CYL experiment.

| Material | Main elements | actual mass [g] | Residual between simulated and experimental decay radiation (GS) [%] | Std [%] | Residual between simulated and experimental neutron projections (NT) [%] | Std [%] | Total residual [%] | Std [%] |
| --- | --- | --- | --- | --- | --- | --- | --- | --- |
| Cu cube | Cu | 8.99 | 1 | 1 | 9 | 7 | 10 | 7 |
| Al-7075 | Cu | 4 |  |  | 12 | 8 | 13 | 8 |
|  | Al | 207 | 2 | 3 |  |  | 14 | 9 |
| Ti6Al4V | Al | 0.21 |  |  | 8 | 6 | 10 | 7 |
|  | Ti | 3.15 | 15 | 11 |  |  | 23 | 13 |
|  | V | 0.14 | 8 | 5 |  |  | 16 | 8 |
| Au bead | Au | 0.14 | 5 | 1 | 33 | 24 | 38 | 24 |
| Ag nugget | Ag | 0.33 | 42 | 13 | 18 | 14 | 60 | 19 |
| Sn plate | Sn | 5.02 | 8 | 4 | 8 | 5 | 16 | 6 |
| Pb plate | Pb | 4.30 | \ | \ | 9 | 8 | ≥ 9 | ≥ 8 |

**Supplementary Table S3.** Summary of the quantified elemental masses of the Kuvera statuette.

| Element | Cu | Sn | Ag | Au | As | Co | Mn | Sb | Cl | Na | Total  (weighted mass) |
| --- | --- | --- | --- | --- | --- | --- | --- | --- | --- | --- | --- |
| Elemental mass [g] | 444 | 74 | 5.36 | 2.30 | 0.40 | 0.18 | 0.003 | 0.13 | 0.08 | 0.20 | 526 (488) |
